# Are video-urodynamics superior to traditional urodynamic studies in changing treatment decision with urinary symptoms?

**DOI:** 10.1080/2090598X.2019.1590518

**Published:** 2019-04-24

**Authors:** Sana H. Ansari, Ayman E. Mahdy

**Affiliations:** a The Christ Hospital, Department of Obstetrics and Gynecology, Division of Female Pelvic Medicine and Reconstructive Surgery, Cincinnati, OH, USA; b Department of Surgery, Division of Urology, University of Cincinnati Medical Center, Cincinnati, OH, USA

**Keywords:** Video-urodynamic, vesico-ureteric reflux, fluoroscopy, reliability, urinary symptoms, urodynamic

## Abstract

**Objective**: To investigate the utility of video-urodynamic studies (VUDS) in patients with various urinary conditions and to evaluate if the addition of fluoroscopic imaging changes the treatment plans one would pursue if urodynamic studies (UDS) alone were performed as VUDS increases cost, radiation exposure, and patient discomfort.

**Patients and Methods**: A retrospective chart review was conducted on all women who underwent VUDS from 2013 to 2015 at one institution. We hypothesised that the addition of the fluoroscopic images would not change the treatment plan. The protocol was conducted in two parts: (i) analysis of the patients’ demographics, history, presentation, and VUDS results; then (ii) comparison of the documented VUDS diagnosis and plan with the theoretical diagnosis and plan of UDS alone.

**Results**: Charts from 156 women were analysed. Fluoroscopic findings impacted the treatment plan in 60 patients. In 38 patients, fluoroscopic findings changed or added to the diagnosis. Vesico-ureteric reflux (VUR) was detected in 16 patients, nine were incidental findings (*P* < 0.001) in which there were no UDS findings of urinary retention (*P* = 0.01) or poor compliance (*P* = 0.02). Fluoroscopic findings of VUR significantly changed diagnosis (*P* < 0.001), but did not significantly change the treatment plan (*P* = 0.09).

**Conclusion**: We conclude that fluoroscopic findings from VUDS do not add to or change the treatment plan. If there is a clinical concern for VUR, UDS with renal imaging would be able to detect findings or potential damage to the upper urinary tract without needing VUDS.

**Abbreviations:** DESD: detrusor–external sphincter dyssynergia; LUT: lower urinary tract; POP: pelvic organ prolapse; PVR: post-void residual urine volume; SUFU: society of urodynamics, female pelvic medicine and urogenital reconstruction; (V)UDS: (video-) urodynamic study; UI: urinary incontinence

## Introduction

Urodynamic studies (UDS) are a common test in patients complaining of LUTS. Although UDS are only one component of a comprehensive evaluation, the findings are often presumed to guide management. However, unlike other studies, such as electrocardiogram, the interpretation of UDS are subjective; one can argue that the role of UDS is to verify and not diagnose lower urinary tract (LUT) conditions when combined with the patient’s verbal concerns and complaints.

Video-UDS (VUDS) include the use of real-time fluoroscopic images. These images allow the addition of some anatomical details of the LUT (and upper urinary tract in cases of VUR) to the functional details that are obtained during the studies. Examples of the relevant anatomical findings found during VUDS are: incompetent bladder neck, intrinsic sphincter deficiency, level of BOO during voiding, bladder diverticula, detrusor–external sphincter dyssynergia (DESD), and VUR. This is in addition to the general findings expected during the scout phase of fluoroscopic studies such as sacral anomalies and radiopaque shadows.

The indications for VUDS are not clear cut (Table 1). There is a paucity of literature surrounding specific indications for VUDS [1]. The AUA and Society of Urodynamics, Female Pelvic Medicine and Urogenital Reconstruction (SUFU) have published guidelines [2] for the use of UDS in LUT conditions and made references to the use of VUDS. They indicate:

- ‘When available, clinicians may perform fluoroscopy at the time of UDS in patients with relevant neurologic disease at risk of neurogenic bladder, in patients with other neurologic disease and elevated PVR or in patients with urinary symptoms’ (Grade C).
- ‘Clinicians may perform VUDS in properly selected patients to localize the level of obstruction, particularly for the diagnosis of primary bladder neck obstruction’ (Expert Opinion).

**Table 1.** Indications for VUDS.

Neurogenic bladder or neurological disease with risk of neurogenic bladder [11]
Unexplained urinary retention [11] or obstructive voiding [1]
Risk factors for poor compliance [11] Prior radical pelvic surgery Pelvic radiation Chronis cystitis Long-term indwelling catheter Long-term anuria
Recurrent UI after prior surgeries [11]
Nocturnal enuresis [11]
Pelvic organ prolapse with voiding dysfunction [11]
Refractory voiding dysfunction, OAB or SUI [11]
History of urological injury or surgery (ureteric re-implantation, renal transplant, pelvic reconstruction) [11]
Abnormal imaging findings (unilateral hydronephrosis or hydroureter) [11]
Congenital genitourinary anomalies [1]

In 2014, Marks and Goldman [1] published on the indications and technique of VUDS, citing that use of fluoroscopy during UDS can aid in the further evaluation of VUR, anatomical variations of the bladder, voiding dynamics in females with pelvic organ prolapse (POP), bladder neck function and coordination during micturition, urethral pathology, DESD, dysfunctional voiding/pelvic floor dysfunction, urinary fistulae, and urinary incontinence (UI).

With the range of use for VUDS being broad and the lack of support in the literature, as well as increased cost, radiation exposure, and patient discomfort, we sought to investigate the utility of VUDS in various urinary conditions. In the present retrospective study, we evaluated conditions in which fluoroscopic images impacted on diagnosis and treatment plan. Additionally, we evaluated if the addition of fluoroscopic imaging on VUDS changed the treatment plan one would pursue if UDS alone were performed.

## Patients and methods

### Sample

A retrospective chart review was conducted on all women who underwent VUDS from 1 December 2013 to 31 December 2015 by one urologist at one institution. A total of 159 charts were reviewed and, of those, three patients were excluded: two where the fluoroscopic findings were accidently revealed, and one who did not have a bladder. Demographic information including past medical, surgical and social history were collected. The charts were reviewed for specific information related to the history of the present illness, medications, review of systems, examination findings, as well as relevant imaging, laboratory results and cystoscopy results done prior to the VUDS. The VUDS results documented in the chart, which included urodynamic diagnosis and fluoroscopic findings, and final treatment plan were also collected for the data-set.

### Study protocol

The study was conducted in two parts. The first part was to focus on the patient’s history, presentation and VUDS results. We did so by evaluating whether the presence of VUR or other findings on fluoroscopy were associated to the patient’s UDS findings, as well as certain aspects of the patient’s history, such as neurological disease or prior genitourinary surgeries.

In the second part of the study, we compared the documented VUDS diagnosis and plan with a theoretical diagnosis and plan of only UDS without fluoroscopic imaging results. In order to do so, each case was presented to the attending urologist, who was blinded to the identity of the patient and time period she was seen by him. The presentation included demographic information; pertinent medical histories; risk factors for urinary issues; the symptoms upon presentation; examination findings, including post-void residual urine volume (PVR); and pertinent ancillary information known prior to the VUDS, such as laboratory results and imaging. The attending was then given the brief UDS report documented in the chart but without fluoroscopic findings. All UDS tracings were made available for review. The attending then gave his urodynamic diagnosis and treatment plan. His diagnosis and plan were compared to the original VUDS (with fluoroscopic findings) diagnosis and treatment plan documented in the chart.

The treatments were categorised as such:

1. No intervention or stop current therapy.
2. Oral medication (β_3_-adrenoceptor agonists, anticholinergics, muscle relaxants, α antagonist, or combination).
3. Minimally invasive (sacral nerve stimulation, chemical denervation of bladder, mid-urethral sling, urethral bulking, urethral dilatation).
4. Major surgery (continent or incontinent diversion with or without anti-incontinence procedure or cystectomy, bladder augmentation).
5. Conservative (pelvic floor physical therapy; fluid and diet management, timed voids; pessary; indwelling catheter, clean intermittent catheterisation, suprapubic tube).

We hypothesised that the addition of fluoroscopic findings would not change the treatment plan. The primary outcomes of the study included:

- Frequency of fluoroscopic images impacting on treatment plan.
- Frequency of fluoroscopic images impacting on diagnosis.
- Frequency of incidental VUR found on VUDS.

Secondary outcomes included:

- Whether the treatments for the VUDS fell within the standard of care for each disease state as defined by the AUA guidelines.
- Whether the VUDS performed was done within the defined criteria to justify such testing based on the AUA/SUFU guidelines or common practice if guidelines did not exist.

### Statistical analysis

Descriptive statistics were performed using Microsoft EXCEL. The Fisher’s exact test and chi-squared test were used as appropriate to identify differences across study variables using STATA version 14 (StataCorp, College Station TX, USA). Cohen’s κ test was used for evaluation of concordance. Statistical significance was defined as a *P* < 0.05.

## Results

### Sample

The demographics of the 156 women who had VUDS performed are summarised in Table 2. The mean (range) age was 54 (17–84) years; and nearly all were Caucasian. Most of the patients had neurological disease (74%) and were currently or had previously tried another therapy for their urinary issues (70%). Of the 116 patients who had neurological disease (Table 3), the top three complaints of urinary issues were:

1. Daytime frequency (*n* = 98, 63%)
2. Urgency UI (*n* = 90, 58%)
3. Nocturia (*n* = 68, 43.5%)

**Table 2.** Demographics of study sample.

Demographic	*n/N* (%)
Neurological disease	116/156 (74)
Pelvic radiation	10/156 (6.4)
History of apical and/or anterior wall prolapse repair	18/156 (11.5)
History of mid-urethral sling or urethropexy	28/156 (18)
Urological surgery	35/156 (22.4)
Currently on or has failed prior UI therapy	109/156 (70)

**Table 3.** Types of neurological disease within the cohort.

Disease	*n/N* (%)
Multiple sclerosis	45/116 (38.7)
Parkinson’s disease	5/116 (4)
Spinal cord injury	6/116 (5)
Diabetes mellitus	22/116 (19)
Spinal cord disease	21/116 (18)
Spinal surgery	12/116 (10)
Cerebral disease	17/116 (14.6)
Seizure disorder	7/116 (6)
CNS autoimmune disease	1/116 (0.9)

Of the 156 patients, 29 did not have a vaginal examination documented and 59 patients were found to have an abnormal finding on examination, most commonly moderate-to-severe vaginal atrophy (*n* = 17) and Stage ≥II apical/anterior vaginal wall prolapse (*n* = 10). In all, 54 patients (34.6%) had a documented elevated PVR.

### Analysis of VUDS

Tables 4 and 5 summarise the UDS and fluoroscopic findings from the VUDS. Looking at fluoroscopic and UDS findings associated with pertinent history, the finding of VUR was not significantly associated with presence of neurological disease, prior pelvic irradiation, prior POP repair or an anti-incontinence procedure. However, VUR was significantly associated with prior urological surgery (*P* = 0.006). Poor compliance was only significantly associated with prior pelvic irradiation (*P* = 0.003). Hypo-/acontractile detrusor and urinary retention UDS findings were not associated with the previously mentioned variables. A UDS finding of DESD was significantly associated with fluoroscopic imaging of DESD, where four of 10 cases had abnormal electromyographic and fluoroscopic findings of DESD (*P* < 0.001).

**Table 4.** Urodynamic findings from the VUDS.

Urodynamic finding	*n/N* (%)
Normal study	13/156 (8.3)
DESD	5/156 (3)
Detrusor smooth sphincter dyssynergia	5/156 (3)
Urine retention	36/156 (23)
Poor compliance	11/156 (7)
BOO	9/156 (6)
Stress UI or intrinsic sphincter deficiency	33/156 (21)
Detrusor overactivity ± leak	51/156 (32.7)

**Table 5.** Fluoroscopic findings from the VUDS.

Fluoroscopic findings	*n/N* (%)
Normal imaging	99/156 (63.4)
VUR	16/156 (10)
Filling defect (bladder diverticulum)	14/156 (9)
Detrusor sphincter dyssynergia (any)	4/156 (2.5)
Closed bladder neck	4/156 (2.5)
Christmas tree appearance	1/156 (0.6)
Urethral diverticulum	1/156 (0.6)
Incompletely relaxed urethra	1/156 (0.6)

Fluoroscopic findings impacted the treatment plan in 60 cases either with pertinent negative or positive findings; for instance, a case with poor compliance on UDS and no VUR detected was considered to impact the treatment plan. Of those in which fluoroscopic findings impacted treatment, UDS findings of poor compliance, urinary retention, and VUR were significant (*P* < 0.001); hypo-/acontractile detrusor barely missed significance (*P* = 0.051), and DESD was not significant. Patient history of neurological disease, pelvic irradiation, POP repair, anti-incontinence procedure, and prior urological surgery, were not associated with cases in which fluoroscopic images changed treatment.

In 38 cases, the fluoroscopic findings changed or added to the diagnosis: 12 bladder diverticula, 16 VUR, four DESD, three closed bladder necks during micturition, incompletely relaxed urethra, and three with no DESD on fluoroscopy with active electromyography. A significant association with a diagnosis change was found between fluoroscopic image findings and UDS finding of poor compliance (*P* = 0.002). The most significant fluoroscopic findings changing diagnosis were detection of VUR (*P* < 0.001) and DESD (*P* = 0.007). No significant association was found between those in which the imaging changed the diagnosis and prior history of neurological disease, POP repair, anti-incontinence procedure, and prior urological surgery; prior pelvic irradiation just missed significant association (*P* = 0.051).

Concerning the finding of VUR (*n* = 16), there was no association with any one neurological disease. The only significant UDS finding was poor compliance (*P* < 0.001). Of the 16 patients with VUR detected, two patients had not had renal imaging done previously, nine had findings of a normal urinary system and five had abnormal findings (hydronephrosis). Concerning the nine incidental VURs detected (*P* < 0.001), they did not have UDS findings of poor compliance and urinary retention (*P* = 0.005 and *P* = 0.019, respectively); however, one patient had hypo-/acontracile detrusor (*P* = 0.262). There was no association with neurological disease, pelvic irradiation, POP repair, anti-incontinence procedure, and prior urological surgery. Prior renal imaging findings were significantly associated with incidental finding of VUR on VUDS (*P* = 0.035).

### UDS vs VUDS

When comparing differences between the presented UDS and documented UDS component of VUDS, 69% (*n*= 108) of cases had a different diagnosis. Using Cohen’s κ equation, intra-rater reliability yielded a κ of 0.33, minimal agreement. Partial discordance between the two UDS interpretations for two or more diagnosis was also noted: 56 cases had one discordance, 19 cases had two discordances, and seven cases had three discordances in diagnosis. When comparing UDS to VUDS diagnosis (including fluoroscopic findings), 36.5% (*n* = 57) of cases had a different diagnosis and 47% (*n* = 73) had a different treatment plan switching treatment category. A switch in treatment categories was not associated with the finding of bladder diverticula (*P* = 0.331), VUR (*P* = 0.432), or DESD (*P* = 0.896). The finding of VUR significantly changed diagnosis (*P* < 0.001), but did not significantly change the treatment plan (*P* = 0.091). The fluoroscopic finding of DESD (four patients), had a non-significant impact on diagnosis and treatment plan changes (*P* = 0.336 and *P* = 0.106, respectively).

All but one case did not meet the indications for VUDS (Table 1). All treatment plans for cases documented and during re-presentation met treatment guidelines (Table 6) for the respective urological disorders.

**Table 6.** Appropriate treatment for LUTS and findings.

Disorder	First-line treatment	Second-line treatment	Third-line treatment
OAB [12]	Behavioural therapy; PFPT; bladder retraining	Oral or transdermal anti-cholinergic or β_3_-adrenoceptor agonist	Intradetrusor onabotulinumtoxinA; sacral nerve stimulation; PTNS
SUI [13]	PFPT; UI pessary	Synthetic mid-urethral sling	Pubovaginal fascial sling; urethral bulking agents; laparoscopic suspension (Burch)
Mechanical obstruction [11]	Mid-urethral sling revision; pessary for prolapse	Surgical prolapse repair	
Chronic urinary retention [14]	Catheterisation (CIC preferable); timed voids; α-blocker; antibiotic for UTI; suprapubic tube over indwelling urethral catheter	Sacral nerve stimulation	
Neurogenic voiding dysfunction [11]	DESD – anticholinergic plus CIC; Neurogenic detrusor overactivity – onabotulinumtoxinA plus CIC	Sacral nerve stimulation; PTNS	Augmentation cystoplasty with or without continent stoma; complete urinary diversion, continent or incontinent
Non-neurogenic voiding dysfunction [11]	α-blocker; Pseudodyssynergia – behavioural modification, PFPT, GABA receptor agonist, PO benzodiazepine	Sacral nerve stimulation	
Poor compliance [11]	Anti-cholinergic or β_3_-adrenoceptor agonist medication plus catheterisation (CIC preferable)	Sacral nerve stimulation; onabotulinumtoxinA with CIC	Augmentation cystoplasty with CIC; complete urinary diversion
Impaired detrusor contractility [11]	CIC; double void		Chronic indwelling catheter, suprapubic tube

PFPT, pelvic floor physiotherapy.

## Discussion

The fluoroscopic images from VUDS did not significantly contribute to the treatment plan or change the treatment plan, although they did significantly change or add to the diagnosis. The finding of incidental VUR was significant despite no findings on UDS that indicated a risk of VUR. However, imaging outside of fluoroscopy with VUDS was significantly associated with the finding of VUR on VUDS, notably hydronephrosis. We also found the lack of consistent diagnosis with presentation of the UDS compared to the documented diagnosis of the UDS component of the VUDS. Based on our present results, VUDS was useful in evaluating bladder diverticula, DESD, obstructive voiding, and possibly with history of pelvic irradiation.

In comparing our present findings with the published literature, we find consistent similarities. In a 2004 retrospective study by Soygur et al. [3], 128 children were evaluated with VUDS in order to assess the role of the test in diagnosis and management of voiding dysfunction. The finding of VUR was 10%, a low incidence. They concluded the VUDS did not change management conditions except in those where VUR was detected, understandable for the paediatric population involved. Hoebeke et al. [4] in a 2001 study reported a 15% VUR detection rate with VUDS done in children with non-neurogenic bladder sphincter dysfunction. In our present study, the finding of VUR was also 10% (16 of 156 cases) and it significantly impacted the diagnosis, but not treatment management. In other studies utilising VUDS [5,6], the authors commented on the UDS findings with minimal to no comment on fluoroscopic findings, leaving one to ponder why VUDS was performed over UDS in the first place.

The other interesting finding from our present study was the lack of intra-observer reliability of the UDS components. Prior literature has already shown lacking to moderate inter-observer reliability [7,8] with interpretation of UDS. With a κ value of 0.3 from our present study, the lack of intra-observer reliability for diagnosis paralleled findings from other published literature [9]. A 2009 study by Smith et al. [10] utilised a similar protocol to ours when they compared the UDS interpretation live to the same UDS shown later for re-interpretation by the same urodynamicists. They found a κ value of 0.37 for clinical diagnosis and a κ value of 0.26 for treatment management, highlighting the lack of intra-observer reliability of the UDS.

The strength of our present study was a large sample size with few exclusions. The two-staged protocol was unique, allowing us to expand upon secondary findings that can further contribute to other published findings. We limited our present study to only female patients, allowing for better comparisons and applicability to this particular population. We conducted VUDS according to acceptable indications. Treatment management for real cases and hypothetical treatment for presented cases were also noted to fall within published guidelines, highlighting our evidence-based management of various urological conditions.

The weakness of our present study was its retrospective nature. We limited our present study to only female patients, thus precluding applicability to male patients. The UDS interpretation and re-presentation of UDS portion of the VUDS study could also have added to bias with possible recollection of memorable cases despite the time interval given in between. More power could have swayed near significant *P* values into significance.

## Conclusion

Our present study found that the fluoroscopic findings from VUDS do not add to or change the treatment plan. If there is a clinical concern for VUR, UDS with renal imaging would be able to detect findings or potential damage to the upper urinary tract without the need for subjecting the patient to fluoroscopy with VUDS.
